# Delayed Diagnosis of Sheehan’s Syndrome Following Postpartum Hemorrhage and Emergency Hysterectomy: A Case Report

**DOI:** 10.7759/cureus.102761

**Published:** 2026-02-01

**Authors:** Kahina Dameche, Sherin Shams, Maha S AlMesallam

**Affiliations:** 1 Family Medicine, Primary Health Care Corporation, Doha, QAT; 2 Operations, Health Centre (HC) Al Waab, Primary Health Care Corporation, Doha, QAT

**Keywords:** family medecine, hypopituitarism (hp), hypothyroid, postpartum haemorhage, sheehan’s syndrome

## Abstract

This case report describes a 50-year-old woman diagnosed with Sheehan’s syndrome, a rare form of hypopituitarism resulting from severe postpartum hemorrhage during a cesarean section in 2019, which led to cardiac arrest and emergency hysterectomy. Despite experiencing persistent fatigue, muscle pain, constipation, and mood changes for five years, her symptoms were repeatedly misattributed to anxiety, anemia, and vitamin D deficiency. A detailed review of her obstetric history, combined with endocrine testing showing central hypothyroidism, adrenal insufficiency, and low IGF-1, led to the correct diagnosis. Initiation of glucocorticoid and thyroid hormone replacement resulted in significant clinical improvement. This case highlights the importance of considering Sheehan’s syndrome in women with nonspecific chronic symptoms and a history of severe obstetric hemorrhage to enable timely diagnosis and treatment.

## Introduction

Sheehan’s syndrome, or postpartum pituitary necrosis, is a rare but potentially fatal endocrine disorder that results from ischemic infarction of the anterior pituitary gland following severe postpartum hemorrhage (PPH) [1,2]. During pregnancy, the pituitary gland undergoes significant physiological hypertrophy, which increases its vulnerability to hypoperfusion in the event of major blood loss or hypotension [3]. The resulting hypopituitarism often includes deficiencies in key pituitary hormones such as adrenocorticotropic hormone (ACTH), thyroid-stimulating hormone (TSH), gonadotropins, and prolactin [4].

The condition frequently remains undiagnosed for years due to its subtle and gradual onset. Its clinical manifestations, such as chronic fatigue, severe lethargy, muscle pain, constipation, and mood disturbances, are often nonspecific and misattributed to more common conditions like depression, anemia, or perimenopausal symptoms [5,6]. In many cases, early postpartum signs such as lactation failure or amenorrhea are overlooked, and the diagnostic delay can extend for years, particularly in low-resource or fragmented healthcare settings [6].

This highlights the critical role of primary care physicians in recognizing and diagnosing Sheehan’s syndrome. A high index of suspicion, especially in patients with a history of severe PPH, is essential. Early diagnosis and appropriate hormone replacement therapy can be life-saving and significantly improve long-term quality of life [2,5].

## Case presentation

A 50-year-old female presented to the Family Medicine clinic with a five-year history of progressive fatigue, severe lethargy, diffuse muscle pain, chronic constipation, low mood, and decreased exercise tolerance. She reported multiple prior evaluations at different healthcare facilities, where her symptoms had been attributed to anxiety, vitamin D deficiency, and anemia. However, her condition continued to deteriorate, prompting a more thorough reassessment.

On detailed review of her medical history, the patient recalled a severe postpartum hemorrhage following a cesarean section five years ago. During that delivery, she experienced cardiac arrest, underwent an emergency hysterectomy, and required more than 70 units of blood transfusion. She also reported failure to lactate postpartum and the complete cessation of menses (secondary amenorrhea) since that event.

On examination, she appeared generally well, with stable vital signs. Her weight was recorded at 74-78 kg across visits. There were no acute findings, but her presentations, like lethargy, constipation, and muscle pain, raised suspicion for central hypothyroidism and possible hypopituitarism. Laboratory testing revealed a critically low free thyroxine (FT4) level with an inappropriately normal thyroid-stimulating hormone (TSH), suggesting central hypothyroidism (Table 1). Morning serum cortisol was also low, supporting adrenal insufficiency. Further endocrine work-up showed a low insulin-like growth factor-1 (IGF-1) level of 22.7 µg/L and a normal adrenocorticotropic hormone (ACTH) level (Table 1).

**Table 1:** Laboratory results for before and after diagnosis ACTH: Adrenocorticotropic Hormone, TSH: Thyroid-Stimulating Hormone, FSH: Follicle-Stimulating Hormone, IGF-1: Insulin-Like Growth Factor 1 Empty or unavailable data cells have been merged where possible; if merging was not feasible, they are marked with a single hyphen (-).

Laboratory Tests	Before diagnosis	After diagnosis & Treatment	Reference values (unit)
Hypothalamic-pituitary-adrenal axis			
Cortisol (AM)	35.3	60.6	138 – 689 (nmol/L)
ACTH	-	26.8	7.2-63.3 (pg/mL)
Hypothalamic-pituitary-thyroid axis			
Free T4	6.2	13.7	11.0-23.3 (pmol/L)
TSH	2.83	0.98	0.30-4.20 (mIU/L)
Hypothalamic-pituitary-gonadal axis			
FSH	7.8	-	in postmenopausal women: 26 - 135 (IU/L)
Hypothalamic-pituitary-somatotropic axis			
IGF-1	22.7	-	69.0-214.0 (ug/L)
Hypothalamic-pituitary-prolactin axis			
Prolactin	433	-	102-495 (mIU/L)

Based on her obstetric history and biochemical findings, a diagnosis of hypopituitarism secondary to Sheehan’s syndrome was made. The patient was started on hydrocortisone 15 mg in the morning and 5 mg in the afternoon, with advice to carry additional doses for stress dosing. One week later, she reported improvement in fatigue and energy levels. Levothyroxine 50 mcg daily was then initiated after glucocorticoid coverage was established.

She continues to follow up with the endocrinology team for ongoing management and is planned for further pituitary imaging to assess structural changes.

## Discussion

Sheehan’s syndrome is an uncommon endocrine disorder resulting from ischemic pituitary necrosis following severe postpartum hemorrhage, leading to varying degrees of hypopituitarism [7]. Although its incidence has decreased significantly in developed countries due to improved obstetric care, it remains a pertinent clinical concern in low-resource settings and among patients with massive PPH [8,9]. The hallmark of the syndrome is the loss of anterior pituitary function, which may manifest months to years after the initial insult, often causing a delay in diagnosis [10].

In this case, the patient’s symptoms, fatigue, lethargy, muscle pain, constipation, and mood disturbances, were initially misdiagnosed as anxiety, vitamin D deficiency, and anemia, a scenario frequently reported in the literature [11]. Such nonspecific presentations underscore the challenge in diagnosing Sheehan’s syndrome, particularly when the connection to obstetric history is not actively sought. Chronic symptoms post-PPH should prompt clinicians to evaluate pituitary function thoroughly.

The biochemical findings of low free thyroxine with an inappropriately normal TSH, low cortisol levels, and low IGF-1 confirm central hypothyroidism and adrenal insufficiency characteristic of hypopituitarism in Sheehan’s syndrome [12]. Mild hyperprolactinemia, as seen in this patient, may reflect stalk effect or partial pituitary damage [13]. Early diagnosis is essential because untreated hypopituitarism can result in severe morbidity and mortality, including adrenal crisis [4].

Management relies on lifelong hormone replacement therapy tailored to the deficient axes. Glucocorticoid replacement must precede thyroid hormone initiation to prevent precipitating an adrenal crisis [14]. This case also highlights the importance of patient education regarding stress dosing during illness or surgery.

Recent studies emphasize the role of MRI in confirming pituitary atrophy or infarction, although imaging findings may vary depending on the timing after the hemorrhagic event [15]. Continued endocrinology follow-up is necessary to monitor therapy effectiveness and adjust hormone dosages.

In summary, Sheehan’s syndrome should remain a differential diagnosis in women presenting with nonspecific symptoms and a history of severe PPH. Greater awareness among primary care providers and timely referral to endocrinology can improve outcomes.

## Conclusions

This case of a 50-year-old female who developed Sheehan’s syndrome following severe postpartum hemorrhage and emergency hysterectomy illustrates the often subtle and delayed presentation of this condition. Sheehan’s syndrome remains an important yet frequently underrecognized cause of hypopituitarism in women with a history of significant obstetric blood loss. The nonspecific and insidious nature of its clinical presentation frequently leads to delayed diagnosis and mismanagement. This case underscores the critical need for heightened clinical vigilance and thorough endocrine evaluation in patients presenting with chronic fatigue, hypothyroid symptoms, and a relevant obstetric history. Early identification and prompt initiation of appropriate hormone replacement therapy are essential to prevent life-threatening complications and improve quality of life. Continued multidisciplinary follow-up is crucial for optimal long-term management. Long-term management of Sheehan syndrome requires lifelong hormone replacement, primarily targeting thyroid, adrenal, and gonadal function, with growth hormone added if needed.
